# Global Bang for the Buck: Cutting Black Carbon and Methane Benefits Both Health and Climate

**DOI:** 10.1289/ehp.120-a245b

**Published:** 2012-06-01

**Authors:** Bob Weinhold

**Affiliations:** Bob Weinhold, MA, has covered environmental health issues for numerous outlets since 1996. He is a member of the Society of Environmental Journalists.

Black carbon and methane have both been implicated in climate change. They also pose more direct human health threats, with black carbon constituting one component of fine particulate matter (PM_2.5_) and methane acting as a precursor of ground-level ozone. An international team of researchers analyzed 14 control measures for human-caused emissions of black carbon and methane to investigate health benefits that might occur in tandem with actions to help mitigate climate change in the next 20–40 years [*EHP* 120(6):831–839; Anenberg et al.].

Black carbon and methane are attractive climate-change mitigation targets because they are relatively short-lived in the atmosphere compared with carbon dioxide, with emission restrictions leading to fairly rapid benefits. Some of the primary sources of methane are fossil-fuel extraction, landfills, livestock, rice production, and wastewater treatment. Among major sources of black carbon are many types of incomplete combustion, including that tied to transportation, industry, housing, and burning of biomass. The 14 measures target sources such as fossil-fuel operations, vehicle emissions, landfill gas, sewage, agriculture, brick kilns, and biomass-fueled cook stoves.

Using published emissions scenarios as a basis for their calculations, the investigators estimate that if all 14 measures were fully implemented by 2030, average global population-weighted surface concentrations of PM_2.5_ could be reduced by 23–34% and ozone could drop 7–17% within the same period. That could prevent anywhere from 640,000 to 4,900,000 premature deaths annually, or about 1–7% of all deaths estimated to occur with the projected global population of 8.4 billion. The estimated health benefits would be due almost entirely to the black carbon control measures—with their attendant reductions in PM_2.5_, organic carbon, and non-methane ozone precursors—and would occur mostly in Asia and portions of Africa. Climate-change mitigation effects could have their own health benefits, although the authors didn’t attempt to calculate these. The authors accounted for just outdoor exposures from biomass-fueled cook stoves, not the well-documented health benefits of avoided indoor exposures; adding that information with a geographically appropriate distribution likely would substantially increase the number of avoided premature deaths.

The researchers used two atmospheric models, each of which predicted substantially different air-quality responses to the 14 measures and incorporated a wide range of data from numerous epidemiological studies as factors in their equations. Uncertainties and variations in the models and data suggest the number of averted premature deaths could be substantially higher or lower. The researchers didn’t address the difficulties or costs of implementing the 14 measures but did note that some related efforts are already under way on many continents, including adoption of European vehicle-emission standards and reductions in cook-stove emissions in some developing countries.

